# Successfully Improving Ocular Drug Delivery Using the Cationic Nanoemulsion, Novasorb

**DOI:** 10.1155/2012/604204

**Published:** 2012-02-27

**Authors:** Frederic Lallemand, Philippe Daull, Simon Benita, Ronald Buggage, Jean-Sebastien Garrigue

**Affiliations:** ^1^Research and Development Department, Novagali Pharma SA, 1 rue Pierre Fontaine, 91058 Evry Cedex, France; ^2^The Institute for Drug Research, School of Pharmacy, The Hebrew University of Jerusalem, POB 12065, 91120 Jerusalem, Israel

## Abstract

Topical ophthalmic delivery of active ingredients can be achieved using cationic nanoemulsions. In the last decade, Novagali Pharma has successfully developed and marketed Novasorb, an advanced pharmaceutical technology for the treatment of ophthalmic diseases. This paper describes the main steps in the development of cationic nanoemulsions from formulation to evaluation in clinical trials. A major challenge of the formulation work was the selection of a cationic agent with an acceptable safety profile that would ensure a sufficient ocular surface retention time. Then, toxicity and pharmacokinetic studies were performed showing that the cationic emulsions were safe and well tolerated. Even in the absence of an active ingredient, cationic emulsions were observed in preclinical studies to have an inherent benefit on the ocular surface. Moreover, clinical trials demonstrated the efficacy and safety of cationic emulsions loaded with cyclosporine A in patients with dry eye disease. Ongoing studies evaluating latanoprost emulsion in patients with ocular surface disease and glaucoma suggest that the beneficial effects on reducing ocular surface damage may also extend to this patient population. The culmination of these efforts has been the marketing of Cationorm, a preservative-free cationic emulsion indicated for the symptomatic treatment of dry eye.

## 1. Introduction

Ophthalmic diseases are most commonly treated by topical eye-drop instillation of aqueous products. These formulations, however, raise technical problems (e.g., solubility, stability, and preservation) and clinical issues (efficacy, local toxicity and compliance). Conventional aqueous solutions are limited to water-soluble molecules and by the fact that within two minutes after instillation over 80% of the product is eliminated via the nasolacrimal drainage system limiting ocular penetration of the drug to less than 1% of the administered dose [1]. Consequently, pharmaceutical companies have been faced with the challenge of developing a formulation for topical administration which would expand the range of potential active ingredients, remain longer on the ocular surface, and provide sustained therapeutic concentrations in addition to meeting the regulatory criteria for approval. The main challenges in ocular drug delivery and key considerations to develop an ophthalmic preparation are listed in Table 1.

Nanotechnologies are currently considered the best solution to improving the ocular delivery of ophthalmic drugs even though products reaching the market using nanotechnologies are still rare [2]. Some reasons for this are that most of the nanosystems, even the pharmaceutically efficient ones, have encountered technical issues such as stability of colloidal systems [3], requirement for new excipients or use of organic solvents noncompliant to regulatory standards, unknown or unacceptable toxicity profiles [4], or unique scale-up and manufacturing requirements.

Notwithstanding, nanotechnology remains a promising approach for ophthalmic drug delivery. Compared to currently available approaches for administering eye drops, nanosystems with bioadhesive properties (e.g., cationic nanoemulsions) are more efficient at delivering the appropriate concentrations of bioactive molecules to the eye. The mechanism underlying the bioadhesiveness of nanosystems is an electrostatic interaction which prolongs the residence time on the ocular surface [5]. To create an electrostatic interaction with the negatively charged cells of the ocular surface, the vector should be positively charged. This is the advantage of the Novasorb cationic nanoemulsion technology.

The aim of this article is to describe the development of the cationic nanoemulsion technology from bench to patients. The first stage of development after an initial proof-of-concept carried out at the University of Jerusalem was to formulate the nanoemulsion with a cationic agent, an oily phase and surfactants compliant with international pharmacopeias (i.e., US and EU pharmacopeias). The objective was to provide a stable and sterile cationic nanoemulsion loaded with an active ingredient approvable by the regulatory agencies. The completion of a full preclinical package and clinical trials in patients with ocular surface disease has led to the successful launch of the first product based on the cationic nanoemulsion technology.

## 2. Cationic Nanoemulsion for Ocular Delivery

As the neuroretina is an extension of the central nervous system, the external eye and its adnexa are designed to protect the internal ocular structures, particularly from harmful chemicals [6]. The first ocular barrier is the eyelid which acts as a shutter preventing foreign substances from contact with the ocular surface. The second barrier is the tears which are continuously secreted to wash the ocular surface of exogenous substances. Hence, the tears are mainly responsible for the short residence time and low absorption of drugs applied topically to the eye. The last protective ocular barrier is the cornea. The neuronal system of the cornea is able to detect changes in pH and osmolality which can induce reflex blinking and tearing. Also, the cornea forms a tight structural barrier made of three different tissue layers with alternating hydrophilic and lipophilic properties to prevent the intraocular absorption of unwanted substances [7].

Many attempts have been made to prolong the exposure time of topically applied ocular treatments and to improve their bioavailability, therapeutic efficacy, or patient compliance by reducing the number of required administrations [8–10]. Hydrogels, now widely used in the ophthalmic pharmaceutical industry, have enabled, for example, a decrease in the frequency of timolol administrations from two instillations daily to only one. Several excipients with either viscosifying or bioadhesive properties are commonly used (carbopol gels, cellulose derivatives, dextran, gelatin glycerin, polyethylene glycol, poloxamer 407, polysorbate 80, propylene glycol, polyvinyl alcohol, polyvinyl pyrrolidone) to prolong the ocular residence time. The use of such excipients, however, remains applicable to only hydrophilic drugs and the advantage of increasing the viscosity must be balanced against the potential disadvantage of inducing ocular disturbances due to the blurring of vision as a result of a change in the refractive index on the ocular surface. Furthermore, other disadvantages of higher viscosity are that more viscous solutions do not easily exit from the bottle tip and may impose limits to the sterilization options during manufacturing. Most recently, sophisticated approaches like punctal plugs with active ingredient [11], contact lens-releasing glaucoma medications, and injectable biodegradable micro- and nanoparticles were proposed but are today at too early a stage to be available to patients [8].

In addition to the challenges of increasing exposure, numerous lipophilic and poorly water-soluble drugs have become available in recent years that could be applicable to the treatment of a variety of ocular conditions. These drugs represent a formulation challenge for pharmaceutical scientists because of aqueous solubility limitations. Dosage forms for topical ocular application of lipophilic drugs include oily solutions, micellar solutions, lotions, ointments, and suspensions. The ocular administration of such dosage forms is not only uncomfortable for the patient but also of limited efficacy. Despite a large variety of submicron-sized colloidal carriers in the ophthalmic drug delivery field, nanoparticles and liposomes attract most of the attention since they appear to have the potential to yield greater efficacy over existing formulations [12, 13].

In the last decade, oil-in-water-type lipid emulsions, primarily intended for parenteral applications, have been investigated and are now being exploited as a vehicle to improve the ocular bioavailability of lipophilic drugs [14, 15]. Among these, nanoemulsions are considered excellent alternative formulations to deliver lipophilic drug substances to the eye. Emulsions provide a high encapsulation rate, an enhanced stability of the active ingredient, and enhanced ocular penetration. The first marketed ophthalmic emulsion drug product was Restasis (Allergan), a preservative-free anionic emulsion of cyclosporine A (CsA) at 0.05% indicated to increase tear production in patients whose tear production is presumed to be suppressed due to ocular inflammation. Although approved by FDA in 2002, Restasis was never accepted by European authorities. Other emulsion-based eye drops available on the US market are artificial tears (Soothe (Bausch & Lomb) and Refresh Endura (Allergan)). Other ophthalmic nanoemulsions are under development and among them are the products resulting from the Novasorb technology, originated from work at the Hebrew University of Jerusalem by Professor Simon Benita and developed by the French pharmaceutical company Novagali Pharma.

**Figure 1:**
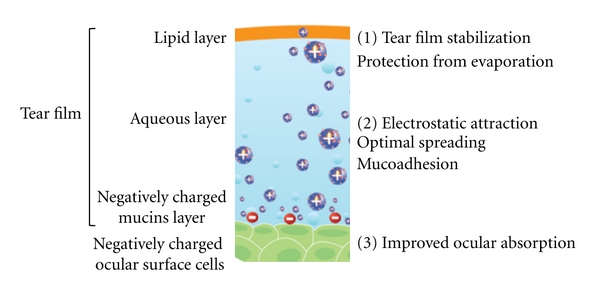
Cationic nanoemulsion interacting with negatively charged corneal cells. The effects of the cationic emulsion are (1) to bring lipids to stabilize the tear film, (2) to interact electrostatically with mucins, and (3) to improve ocular absorption.

The Novasorb technology platform is based on the cationic nanoemulsion approach. The overall Novasorb strategy exploits the fact that the corneal and conjunctival cells and the mucus layer of glycosyl amino glycans lining the ocular surface are negatively charged at a physiological pH [16]. When applying a positively charged formulation to the eye it is likely that an electrostatic attraction will occur prolonging the residence time of the formulation on the ocular surface (Figure 1). In addition, the nanosize of the oil droplets creates a huge contact surface with the ocular surface cells enabling enhanced absorption. This approach was primarily conceived for oral administration [17] and it was adapted a few years later to ocular delivery by Klang et al. [18] to deliver indomethacin and Abdulrazik and coworkers [19] who intended to deliver cyclosporine A.

The potential of cationic emulsions for ophthalmic drug delivery was rapidly seen to offer advantages over the existing topical drug delivery vehicles [20–22]. However, this drug delivery approach was not exempt of hurdles and technology challenges particularly in the formulation phase as we will see further. During the development (from nonclinical to clinical), the products had to go back to the formulation stage to optimize their physicochemical properties due to stability, toxicity, or pharmacokinetic issues. Up to three generations of cationic nanoemulsions were then tested and patented over the 10 years of development [23–25].

## 3. Formulation Development

### 3.1. Cationic Agent

The surface charge of the nanoemulsion is defined by the zeta potential. It corresponds to the electric potential surrounding the oil nanodroplet at the plane of hydrodynamic shear. It is measured by electrophoretic mobility. The latter depends on the nature of the cationic agent, its concentration and the electrolyte environment of the oil nanodroplets. In addition to increasing the residence time on the negatively charged ocular surface, the positive charge of the cationic agent contributes to the stabilization of the emulsion by creating an electrostatic repulsion between the oil droplets of the nanoemulsion [26]. Evidence that the specific nature of the cationic molecule may be responsible for improved uptake properties was supplied by Calvo et al. who showed that two different types of cationic indomethacin loaded nanocapsules (coated with poly-L-lysine or chitosan) resulted in completely different drug kinetics profiles [27]. Therefore, the cationic agent selected needs to be carefully considered prior to starting pharmaceutical development as the success of the formulation is highly dependent upon the choice of the cationic agent as will be discussed further.

Novagali showed that below a zeta potential of +10 mV, nanoemulsions could not be autoclaved without destabilizing the oil droplets. Therefore, the first challenge of the Novasorb technology was to make a cationic emulsion with a zeta potential sufficiently high to stabilize the nanoemulsion, yet with a cationic surfactant concentration as low as possible to avoid compromising the safety of the nanoemulsion. The optimal range for the zeta potential was demonstrated to be between +20 mV and +40 mV. Review of the literature revealed that of the numerous cationic agents described (Table 2) most of them are surfactants, indeed the positively charged region of the molecule does not enter the oil core of the droplet but instead remains at the surface, rendering them very useful for emulsions. Unfortunately, very few are listed in pharmacopeias or accepted for ophthalmic products due to stability or toxicity issues.

Compared to anionic and nonionic surfactants, cationic surfactants are known to be the most toxic surfactants [28]. Therefore, in order to develop the Novasorb technology it was necessary to find an appropriate cationic surfactant which would provide a sufficiently high cationic charge, have a low toxicity, and conform to regulatory standards.

Stearylamine is one of the most widely used cationic lipids in the academic world especially for the manufacture of cationic liposomes [29] or cationic emulsions [19]. However, since this primary amine is very reactive towards other excipients and active ingredients and not described in any pharmacopeias, it was not a reasonable choice for pharmaceutical development. Oleylamine is another cationic lipid that has been used to manufacture ophthalmic emulsions [30], but this lipid also has stability concerns due to its primary amine function and the presence of an unsaturated site in the aliphatic chain.

Other cationic molecules usually used for DNA transfection are also frequently used for the formulation of cationic drug delivery systems: poly(ethylenimine) (PEI) and poly-L-lysine (PLL). PEI is an organic polymer that has a high density of amino groups that can be protonated. At physiological pH, the polycation is very effective in binding DNA and can mediate the transfection of eukaryotic cells [31]. It has been used as a cationic agent in micelles [32], nanoparticles [33], albumin nanoparticles [34], liposomes [35], and nanosized cationic hydrogels [36]. However, while some authors claim this polymer to be safe some others such as Hunter [37] have reported PEI to be extremely cytotoxic. PLL is a polymer made of several lysines (amino acid). Lysine possesses a NH_2_ function which is ionized at a physiological pH conferring several cationic charges to that polymer. It is sometimes used as cationic agent in drug delivery systems such as microparticles [38]. However, toxicity has been reported [39], and this polymer is not authorized for use in ophthalmic formulations.

Cationic lipids, DOTAP (N-(1-(2,3-dioleoyloxy) propyl)-N,N,N trimethylammonium) chloride and DOPE (dioleoyl phosphatidylethanolamine), represent another potential class of cationic agents. These are amphiphilic molecules with a fatty acid chain and a polar group bearing a cationic charge. Their main advantage is that they are biodegradable and well tolerated. DOPE, which also harbors a negative charge, is a neutral “helper” lipid often included in cationic lipid formulations like cationic nanoemulsions [40]. Cationic solid lipid nanoparticles were successfully made with DOTAP to transport DNA vaccines [41]. Hagigit and colleagues [42, 43] showed that using DOTAP was better than the seminatural lipid oleylamine to make stable cationic emulsions. Moreover, DOTAP cationic emulsion enhanced the penetration of antisense oligonucleotides after either topical ocular instillations or intravitreous injection. But like most of the seminatural lipids, these agents are chemically unstable and need to be stored at −20°C, thus drastically limiting their industrial use.

The primary limiting factors against the use of the previously cited cationic agents in the Novasorb technology, even though they showed potential in the formulation of cationic drug delivery systems, is that (1) they are not listed in US and EU pharmacopeias or (2) their toxicity on the ocular surface has not been well documented, and (3) none of these cationic agents has been successfully commercialized in a pharmaceutical product. Consequently, Novagali chose to limit its search for the appropriate cationic agent among those already registered, used in ophthalmic products, or compliant to pharmacopeias.

Other excipients previously accepted by health authorities were then considered. Quaternary ammoniums usually used as preservatives have surfactant properties and the potential to give a cationic charge to the nanoemulsions. These agents include cetrimide, benzalkonium chloride, benzethonium chloride, benzododecinium bromide, and cetylpyridinium. As preservatives these products protect against infectious contaminants by electrostatically binding to the negatively charged surface of bacteria and mycoplasma and disrupting their cell membranes. The disadvantage of quaternary ammoniums is that their effect on cell membranes is not limited only to microorganisms but they are also capable of injuring epithelial cells lining the ocular surface by the same mechanism of action. It was consequently not obvious to foresee these molecules as cationic agents, therefore, quaternary ammoniums were not initially considered for use in emulsions. In 2002, Sznitowska revealed findings that the preservative efficacy of this class of surfactants was diminished or neutralized in the presence of emulsions [44]. Part of the quaternary ammonium is bound to the emulsion, resulting in the presence of less free surfactant molecules in the aqueous phase to exert their antimicrobial action, and, consequently, their toxic effect on the ocular surface epithelia. Novagali Pharma exploited this physicochemical property to make a new type of cationic nanovector using benzalkonium chloride (BAK) and cetalkonium chloride (CKC) as cationic agents. CKC is a highly lipophilic (log⁡*P* = 9.5) component of BAK. It is hence mostly included in the oily phase providing a higher zeta potential on surface of the oil droplets while leaving relatively no free molecules to induce ocular surface toxicity. BAK (and CKC as a component of BAK) has been routinely used as a preservative in other marketed eye drop solutions (e.g., BAK is used in Xalatan) and is accepted as compliant with regulatory requirements for ophthalmic products. These excipients used in lower concentrations as cationic agents in emulsions have been demonstrated to be safe for the eye as we will see in the toxicology chapter of this article. More importantly, the use of BAK and CKC as cationic surfactants only in emulsions are now protected by several granted and pending European and US patents (e.g., EP1655021 [25], EP1809237 [45], EP1809238 [46], and EP1827373 [47] which are granted).

### 3.2. Other Formulation Issues

Following the choice of the cationic agent, other excipients, that is, nonionic surfactants, osmotic agents, and oils, need to be selected and their appropriate concentration decided (Table 3). The excipients authorized for ophthalmic use are quite numerous and this step of screening was mainly time dependent. An emulsion is a system which is by essence unstable. The stability is further ensured by the combination of excipients with the surfactants; this combination also defines the size of the emulsion. The concentration of surfactants should be a compromise between stability and toxicity. The most commonly used surfactants are poloxamers, polysorbates, cremophors, tyloxapol, and vitamin E TPGS.

To choose the appropriate excipients and their concentration, parameters like the final osmolality and pH of the nanoemulsion need to be considered. The product to be applied on the eye surface should have these parameters close to physiological values. This introduces another difficulty as the buffers and osmotic agents may also hide the surface charge of the cationic nanodroplets and potentially destabilize the emulsion. Normal tears have a pH between 6.9 and 7.5 [48]. The literature indicates that the ocular instillation of 20 *μ*L of a buffered solution at pH 5.5, 0.067 M is quickly brought to pH 6–6.5 in the tears [49]. Furthermore, it is usually known that a low pH is well tolerated if it is rapidly brought back to normal tear pH [50], therefore it can be assumed that buffering is not so important. In the case of Novasorb, the emulsion can be slightly buffered with a tris buffer (Cationorm) or not buffered at all, leaving the natural pH of the mixture. In that case, the tears rapidly restore the physiological pH of the lacrimal film.

Neutral osmotic agents, such as polyols (glycerol, mannitol, or sorbitol) were used. The lipid emulsions more or less physically resemble a simple aqueous-based eye drop dosage forms since more than 90% of the external phase is aqueous irrespective of the formulation composition. The main difference is its visual aspect: a milky white appearance. The final specifications are summarized in Table 4. It should be noted that even though BAK or CKC is present in the product as the cationic agent, the formulations are not preserved [51]. Thus, emulsions are packaged in single use vials filled by the Blow-Fill-Seal technology. Finally, the vehicle typically has a formula as presented in Table 5. Active ingredient is added in the oily phase but some hydrophilic molecules could be added in the aqueous phase to create a combination product.

The size of the oil nanodroplets is of utmost importance as it contributes to the stability of the emulsion and to the ocular absorption. To our knowledge, it has not yet been demonstrated that ocular absorption is correlated to the size of the nanovectors even if it is logical that the smaller the object, the higher the expected uptake. As discussed by Rabinovich-Guilatt et al. [21], there are several mechanisms of absorption of nanoparticles in the cornea. In the case of cationic nanoemulsions, positively charged nanodroplets of oil are not likely to penetrate the cornea as the drops are bound to the negatively charged mucus. Therefore, the delivery of the active ingredient is probably related to a passive diffusion linked to the enhanced retention time.

An additional factor favoring drug absorption is linked to the small size of the nanodroplets, that is, the interfacial area available for drug exchange. If the mean diameter of an oil droplet is 150 nm, and the volume of emulsion administered on the ocular surface is about 30 *μ*L, the number of oil nanodroplets administered is close to 10^10^. Consequently, with such an extraordinarily elevated specific surface of exchange (almost 1,000 mm^2^) the diffusion of the active ingredients to the targeted tissues is greatly improved. Thus, a small droplet size of the nanoemulsion should consequently be associated with an improved clinical efficacy of the drug.

**Figure 2:**
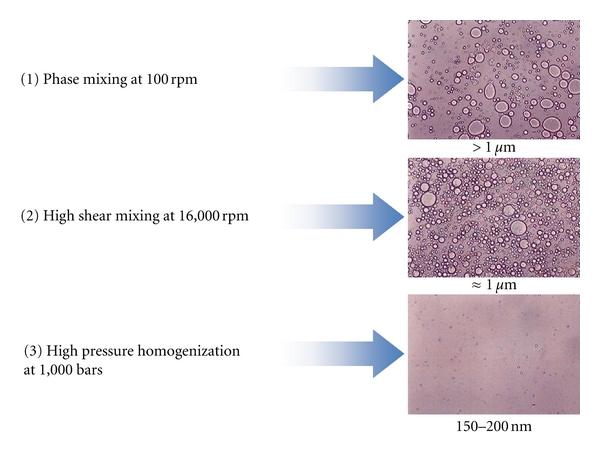
Three manufacturing steps of the process necessary to decrease the oil droplet size of the emulsion. Optical microscopy pictures of the emulsions are presented.

The manufacturing process is a three-step process as described in Figure 2. The first step is a phase mixing under magnetic stirring at 100 rpm for a few minutes followed by a high shear mixing at 16,000 rpm during 10 min at that stage the oil droplets of the emulsion have a size of approximately 1 *μ*m. To reach a submicronic size (150–200 nm) the emulsion is submitted to a high pressure homogenization at 1,000 bars under cooling.

Stable cationic nanoemulsions were selected over hundreds of prototypes after being submitted to screening stress tests (freeze/thaw cycles, centrifugation, and heat test at 80°C). In addition, a deep physicochemical characterization including measurement of pH, osmolality, zeta potential, droplets size, interfacial and surface tension, aspect, and viscosity was systemically performed on prototypes. All these tests are able to discriminate a potential destabilization of the emulsions like creaming, coalescence Ostwald ripening, and phase separation and to set final specifications of the drug product as described in Table 4.

Finally, the product should be sterile. Since the sterilization process can have a major impact on the physical integrity of the emulsion, it should be taken into account at an early stage during the development of the formulation. A sterilizing filtration is not possible for emulsions as it uses a filter with 0.22 *μ*m size pores that can clog during filtration. Aseptic processes are too expensive. The remaining option was heat sterilization; however, this can be performed only on very stable emulsions, and hence the need of a careful choice of the above-mentioned excipients.

### 3.3. Drug Loading

The Novasorb technology platform was ultimately designed to be loaded with active molecules. Emulsions are clearly adequate for lipophilic drugs with a log P of 2-3 (P: octanol/buffer pH 7.4 partition coefficient) preferentially nonionizable, and such candidates are numerous. Even so, the cationic emulsion with no active ingredient itself possesses beneficial properties. Its composition comprising oil, water, surfactants, and glycerol reduces evaporation of tears from the ocular surface while lubricating and moisturizing the eye. Altogether the components confer a protective effect by augmenting each layer of the tear film. Based on the inherent properties of the Novasorb technology, restoring the deficient layers of the natural tear film, Cationorm, a preservative-free cationic emulsion containing no active ingredient, has been commercialized globally for the relief of dry eye symptoms (Table 6).

Nearly 40% of new chemical entities have a low aqueous solubility, therefore potential candidates to be loaded into Novasorb [52]. Novagali Pharma incorporated about 40 lipophilic active ingredients of various therapeutic classes (NSAID, SAID, antibiotics, antifungals, etc.) proving the versatility of this emulsion. Herein, we will only focus on the most advanced products. Despite topical administration in solvents yielding poor bioavailability, CsA, a very lipophilic immunomodulatory drug, is widely used by ophthalmologists due to its recognized therapeutic potential for the treatment of ocular diseases (dry eye, allergy, and inflammation) [53]. CsA was considered an excellent initial candidate to evaluate the potential of the Novasorb cationic emulsion to improve the efficacy of an established drug. Therefore, the primary challenge in the development of a cationic emulsion containing CsA was to design the optimal formulation [53] for topical delivery. Today, Novagali Pharma has developed two products based on the Novasorb technology loaded with CsA: Cyclokat for the treatment of dry eye and Vekacia for the treatment of vernal keratoconjunctivitis.

Latanoprost, a lipophilic prostaglandin analogue, is a potent intraocular pressure lowering agent currently marketed as Xalatan (Pfizer,) for the treatment of glaucoma and ocular hypertension. In Xalatan, the active ingredient, latanoprost 0.005%, is solubilized in water by 0.02% of BAK. Despite being the leading antiglaucoma medication, there are two drawbacks of Xalatan that may have impacted its huge commercial success: (1) the formulation was not stable at room temperature necessitating storage at 5°C and (2) BAK in the formulation as a preservative and solubilizing agent causes ocular surface toxicity which probably resulted in decreased compliance. As the patent protecting this molecule is expiring in 2011, there was an opportunity to improve upon the disadvantages of Xalatan. Hence, Novagali launched the development of Catioprost, a preservative-free cationic emulsion loaded with latanoprost for the treatment of elevated intraocular pressure (IOP) while protecting and improving the ocular surface.

## 4. Nonclinical Development

The nonclinical development is divided into the safety evaluation and the pharmacokinetic studies.

### 4.1. Safety

Establishing the safety of the new nanotechnology was an important goal of the nonclinical development program. Toxicity is a major concern in nanotechnology as the behavior of the nano-object is difficult to predict [4]. Therefore, numerous studies were conducted to ensure the ocular safety of the cationic emulsion.

As the active ingredients used in Novagali's emulsions (CsA and latanoprost) are already used in other drug products only the toxicity of the vehicle and the final product was evaluated.

Before the development of Novasorb, preliminary data regarding the ocular safety of some cationic emulsions on the eye were already available [54]. A subchronic toxicity study performed in rabbits demonstrated that a cationic emulsion containing 3 mg/mL stearylamine was found to be safe and well tolerated after repeated topical ocular administrations [54]. In addition, a local tolerance study in rabbit eyes demonstrated that a 1 mg/mL oleylamine ophthalmic emulsion instilled eight times per day for 28 days was relatively well tolerated [21]. These data, even though promising, were not sufficient to support further development as Novasorb utilizes cationic agents (CKC and BAK) that are usually used at higher concentrations as preservatives. The safety profile of Novasorb cationic emulsions using BAK as a cationic agent was thus evaluated in both *in vitro* and *in vivo* models as listed in Table 7.

#### 4.1.1. Safety of Novasorb as Vehicle

During the formulation work, emulsion prototypes were quickly evaluated by the Draize test which, despite a few limitations, allowed the identification of the least irritating nanoemulsion. This test consists of instilling 30 to 50 *μ*L of the product into one eye of 6 New Zealand white rabbits and monitoring to observe any abnormal clinical signs such as redness of conjunctiva, swelling, or increased blinking which may indicate irritation. The test does not give objective values as it is operator dependent but gives a good idea of how the product will be tolerated.

Other *in vitro* and *in vivo* tools were used. In an *in vitro* scrapping assay using human corneal epithelial (HCE) cell monolayers, a cationic emulsion containing 0.02% BAK as a cationic agent was as well tolerated as a phosphate buffered saline (PBS) solution while an aqueous solution of 0.02% BAK revealed toxicity.

An acute toxicity rabbit model was used which allows for the characterization of the mechanism underlying the toxicity observed during the conventional Draize tests [55]. In the experiment, 15 instillations of test eye drops are administered at 5 min intervals, with observations performed over 96 hours. Clinical signs, *in vivo* confocal microscopy, and conjunctival impression cytology were performed to assess the safety profile of the different cationic emulsions with BAK or CKC as the cationic agent. This study demonstrated that cationic emulsions using BAK or CKC as the cationic agent were very well tolerated while the tested 0.02% BAK solution was responsible for corneal epithelial cell death related to the proinflammatory and proapoptotic activity of BAK.

#### 4.1.2. Safety of Novasorb Loaded with Active Ingredients

**Figure 3:**
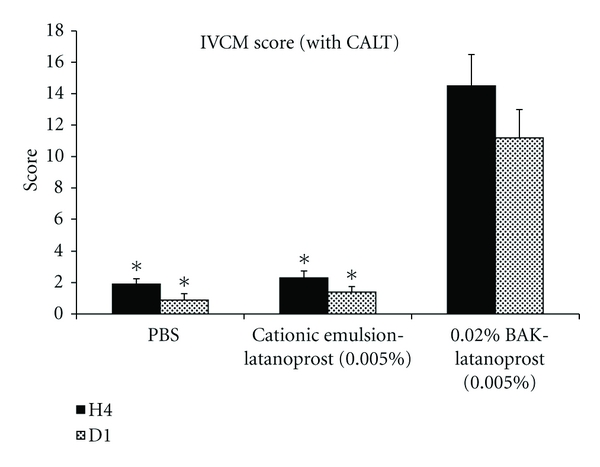
*In vivo* confocal microscopy score of rabbit ocular surface following repeated instillations with Novasorb cationic emulsion of latanoprost. IVCM images of rabbit ocular surface and conjunctiva associated lymphoid tissue (CALT) were used to assess the safety of the cationic emulsion of latanoprost by scoring the alterations observed following repeated instillations. Note that the lower the score the better the tolerance. PBS was used as a negative control. (*) *P* < 0.0001 compared with 0.02% BAK-latanoprost (0.005%). Adapted from Liang et al. [56].

The safety profile of the Novasorb used as a vehicle for lipophilic drugs such as cyclosporine (Vekacia/Cyclokat) and latanoprost (Catioprost) was evaluated in animal models [56]. These studies demonstrated that neither of the two active ingredients (CsA or latanoprost) has an impact on the safety profile of the cationic emulsions as both drug-loaded cationic emulsions were as well tolerated as the cationic emulsion vehicle (Figure 3). For example, in the acute toxicity rabbit model, repeated instillations of Cyclokat/Vekacia (CsA-containing 0.05 and 0.1% CsA cationic emulsions) were as well tolerated as Restasis (0.05% CsA anionic emulsion), and Catioprost (preservative-free latanoprost 0.005% cationic emulsion) was better tolerated than the 0.02% BAK-preserved Xalatan. Local tolerance studies in the rabbit confirmed that chronic instillations (4–6 times daily over 28 days) with Cyclokat/Vekacia and twice daily for Catioprost were well tolerated by the rabbit eyes.

All the previous *in vivo* data were obtained in rabbits with a healthy ocular surface. However, it was of interest to also assess the effect of Catioprost on damaged corneas to more closely mimic the clinical situation experienced when elderly patients are started on glaucoma therapy. For that purpose, a rat model of debrided cornea was used to assess the effect of Catioprost, its emulsion vehicle, and Xalatan (the commercially available product of latanoprost) on the ocular surface healing process. The in vivo data demonstrated that Xalatan delayed corneal healing, while both Catioprost and its cationic emulsion vehicle (without latanoprost) promoted healing of the ocular surface and restored the function of the injured epithelium, thus confirming the better safety profile of the Novasorb cationic emulsions and confirming that Novasorb could hasten the repair of ocular surface damage. Novasorb was hence shown to be safe, but prior to human testing several other studies were necessary to fulfill the various European and American guidelines. These studies cited in Table 7 included *in vitro* evaluation of the cytotoxic potential by indirect contact, a delayed-type hypersensitivity evaluation in the guinea pig, an ocular irritation test in the rabbit (short term: 72 h) following a single application, a determination of the physical compatibility of Novasorb with contact lenses, a 28-day ocular tolerance in the rabbit, an evaluation of the potential to induce delayed contact hypersensitivity (local lymph node assay), an evaluation of the corneal sensitivity following repeated applications in albino rabbits, an evaluation of potential phototoxicity and photoallergy following topical applications in the guinea pig and finally a 6-month ocular toxicity in the dog and rabbit. The description of these entire assays can be found in the various regulatory guidelines.

### 4.2. Proof-of-Concept Studies and Pharmacokinetics

In parallel to ensuring the safety, proof-of-concept studies were performed in order to validate the cationic nanoemulsion technology in the ocular delivery of active molecules.

**Figure 4:**
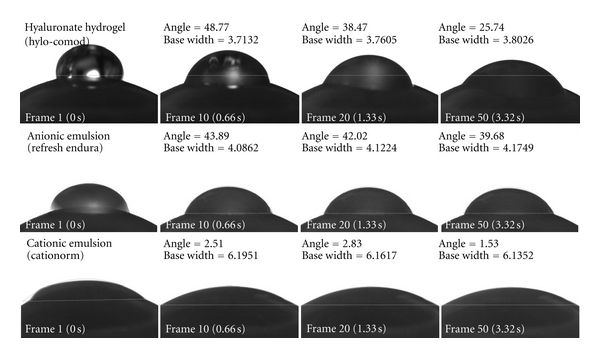
Dynamic contact angle measurement and base width of an eye drop instilled on rabbit eyes. Photos taken at 0, 0.66, 1.33, 3.32 seconds after instillation of hyaluronate hydrogel (Hylo-COMOD), anionic emulsion (Refresh Endura), and cationic emulsion (Cationorm). Contact angle and base width values confirm the optimal and fasted spreading of cationic emulsions compared to anionic emulsions and hyaluronic acid based product.

To assess the effect of the cationic charge on the ocular surface, Novagali Pharma has performed static and dynamic contact angle and surface tension studies on harvested rabbit eyes according to a method adapted from Tiffany [57]. This experiment showed that Novasorb cationic emulsions have a better spreading coefficient on the cornea and conjunctiva than conventional eye drops and anionic emulsions. This improved spreading coefficient leads to better ocular surface wettability. Optimal spreading of the cationic emulsion confers protective filmogenic properties and reduces tear washout.  Figure 4 illustrates the behaviour of the cationic emulsion which spread over the eye very rapidly compared to other formulations. It has been well described that oil-in-water emulsions enhance drug absorption by facilitating corneal or conjunctival absorption or prolonging the contact with the eye, thus improving drug delivery [58].

Early pharmacokinetic studies were performed to evaluate CsA absorption following the application of experimental 0.2% CsA cationic and anionic emulsions [19]. The data demonstrated that the cationic emulsion was almost two-times better at delivering CsA to ocular tissues than an anionic emulsion, even though the latter contained 0.01% BAK and 0.2% deoxycholic acid as a mild detergent that can disrupt cell membranes and serve as a permeation enhancer.

Restasis (Allergan) is an anionic emulsion of CsA (0.05%) that has been shown to readily penetrate ocular tissues without significant systemic passage [59, 60]. Pharmacokinetic (PK) studies designed to evaluate the ocular and systemic CsA distribution following single and multiple dosing with cationic emulsions NOVA22007 (cationic emulsion at 0.05%) or Cyclokat (cationic emulsion at 0.1%), compared to Restasis as a reference, confirmed the beneficial role of the cationic charge in enhancing the ocular penetration of CsA [61] in Novasorb cationic emulsions.

**Figure 5:**
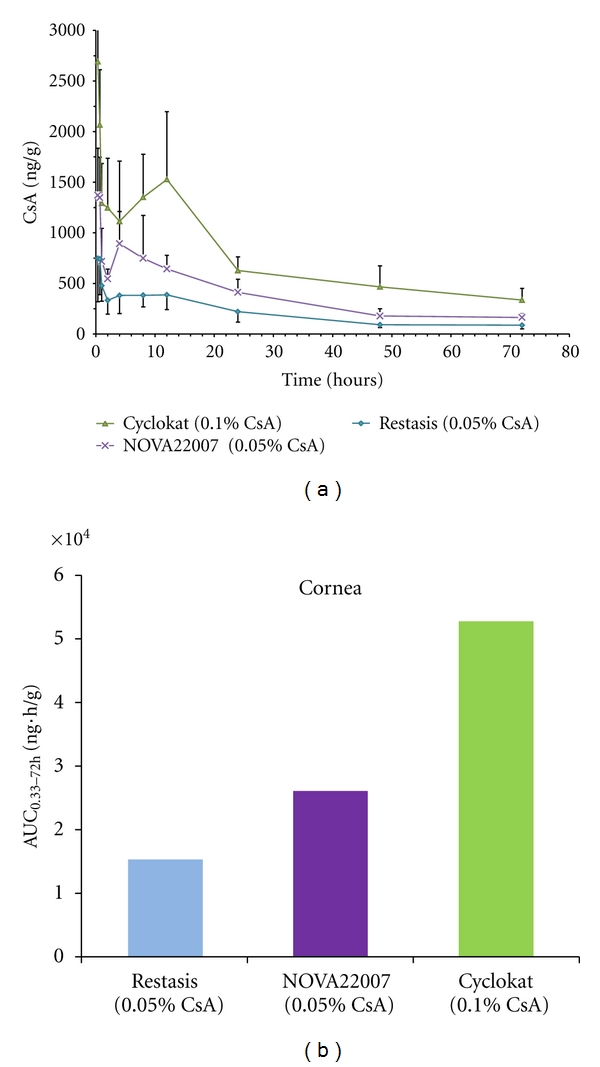
(a) Changes in corneal CsA concentration with time after a single unilateral topical administration in pigmented rabbits. The error bars represent standard errors. (b) Cornea absorption (AUC) following a single instillation in pigmented rabbits.

Single-dose PK data demonstrated that the 0.05% CsA cationic emulsion was more effective than Restasis at delivering CsA to the cornea (Cmax: 1372 versus 748 ng/g; AUC: 26477 versus 14210 ng/g.h, resp.). Furthermore, multiple-dose PK confirmed that there was no systemic absorption, with values below the limit of detection (LOD, 0.1 ng/mL) for the CsA-cationic emulsion (see Figure 5). The use of 3H-CsA also demonstrated that the systemic distribution following repeated instillations was indeed low and comparable for both the CsA-cationic emulsion and Restasis and confirmed that the improved local absorption with the CsA-containing cationic emulsion did not translate into increased systemic CsA levels.

In addition, the electroattractive interactions between the positively charged oil droplets of the cationic emulsion and the negatively charged ocular surface cell epithelia might also explain the 50% lower contact angle observed with cationic emulsions versus anionic (negatively charged) emulsions, and the higher spreading coefficient [18]. A low contact angle, better spreading coefficient, and an increased residence time of the cationic emulsions may all contribute to the better drug absorption of lipophilic drugs solubilized in cationic emulsions.

The cationic emulsions designed for the treatment of dry eye disease (Cyclokat) and vernal keratoconjunctivitis (Vekacia) were not tested in pharmacodynamic models as there are no reliable experimental models for these pathologies. However, pharmacokinetic studies with CsA cationic emulsions in animal models demonstrated (see previous paragraph) that the tissue concentrations of CsA were above the therapeutic concentration (50–300 ng/g of tissue according to Kaswan [62]) in both the cornea and conjunctiva. Therefore, the safety and efficacy of these CsA-containing cationic emulsions were first demonstrated in phase II and III clinical trials (see the following section).

In contrast, the safety and efficacy of Catioprost (preservative-free latanoprost 0.005% cationic emulsion) was initially evaluated in an established cynomolgus monkey model of ocular hypertension [63], and compared to Xalatan. Both latanoprost formulations shared the same efficacy profile, and the intraocular pressure (IOP) reduction lasted 24 h. Additionally, a comparison of the local tolerance of Catioprost and Xalatan following twice-daily repeated instillations in rabbits over a 28-day period revealed, although both products were well tolerated, there was a 42% lower incidence of conjunctival redness in rabbits treated with Catioprost. Overall, the results of the preclinical models suggested that Catioprost appears to be as potent as Xalatan for the reduction of IOP with an improved safety profile.

As listed in Table 8, some pharmacokinetic studies are compulsory prior to human testing. They include the single- and multiple-dose pharmacokinetic studies, the determination of systemic exposure, plus the toxicokinetic studies following repeated instillations. The full nonclinical package gave a high confidence that Novasorb technology alone or loaded with active ingredients was fully safe and could provide high concentration of active ingredient in ocular tissues. The next step of the development was then the clinical evaluation in human.

## 5. Clinical Development

An IND-enabling dossier was prepared allowing for conduct of a first-in-man clinical trial. This dossier was prepared according to guidance received through regulatory interactions with health agencies (FDA, EMA). Indeed, early exchanges with health agencies about technologies are possible to discuss technology specific requirements (efficacy, safety) and anticipated clinical and regulatory development programs.

Table 9 describes the different clinical trials carried out to the evaluate Novasorb technology with or without an active ingredient. The clinical development was first performed with a drug-free cationic emulsion formulation (vehicle). The first clinical trial was carried out with the first generation of the cationic emulsion in 16 healthy volunteers. The safety and tolerance of four-times daily instillations was evaluated over 7 days of treatment. The product was shown to be safe and well tolerated. Since the vehicle harbors intrinsic properties of ocular surface protection, it was then tested in two phase II clinical trials aiming at evaluating the efficacy, tolerance, and safety of Cationorm in patients with mild to moderate dry eye (results are detailed in the next section).

A cationic emulsion containing CsA was subsequently evaluated in patients with either dry eye disease (DED) or vernal keratoconjunctivitis (VKC). Highlights of some clinical results are detailed below in light of challenges faced including efficacy of the “placebo” comparator which was the cationic emulsion vehicle, variability of endpoints, and disconnection between sign and symptoms of ocular surface diseases.

Finally, a phase II program was initiated with Catioprost, the cationic emulsion containing latanoprost. Since the phase II trial is ongoing, no data are available.

### 5.1. Clinical Evaluation of Cationorm

In the 2007 Dry Eye Workshop (DEWS) report, dry eye disease (DED) is defined as a multifactorial disease of the tears and ocular surface that results in symptoms of discomfort, visual disturbance, and tear film instability with potential damage to the ocular surface. Currently, symptomatic treatment with artificial lubricants is the first line of treatment for patients with DED; however, the disadvantage of most conventional artificial tear solutions is that most of the instilled drug is lost within the first 15–30 seconds after installation, due to reflux tearing and the drainage via the nasolacrimal duct. The prolonged residence time of the cationic emulsion on the ocular surface due to the electrostatic attraction between the positively charged lipid nanodroplets and the negatively charged ocular surface and the augmentation of the tear film layers by the oily and aqueous phase of the emulsion suggested that the Novasorb technology could be inherently beneficial for the ocular surface even in the absence of an active ingredient.

Consequently, the ocular tolerance and efficacy of Cationorm, a preservative-free cationic emulsion, were evaluated and compared to Refresh Tears (Allergan) in a one-month, phase II, multicenter, open-label, randomized, parallel-group study enrolling patients with signs and symptoms of mild to moderate DED. Adults with a history of bilateral DED were subjected to a washout period of prior DED treatments during which only artificial tears were allowed. At the inclusion visit patients were randomized to treatment with either Cationorm (*n* = 44) or Refresh Tears (*n* = 35) in both eyes 4 times daily and evaluated at follow-up visits on Day 7 and Day 28. Ocular tolerance and efficacy were assessed at one month. Seventy-nine patients, 86% female with a mean age of 61.6 years, were enrolled in the study. At 1 week and 1 month the mean reduction in individual dry eye symptoms scores and total dry eye symptoms scores were greater in the Cationorm than Refresh Tears treated patients (36% versus 21% at Day 7, and 49% versus 30% at Day 28, resp.) demonstrating that DED symptoms improved better with Cationorm. While the global local tolerance was perceived similarly with both treatments, the study investigators rated the overall efficacy of Cationorm statistically significantly better than Refresh Tears (*P* < 0.001). Additionally, Cationorm-treated patients experienced greater improvements from baseline compared to Refresh Tears-treated patients for the Schirmer test (1.88 versus 1.27 mm) and corneal fluorescein staining (−0.61 versus −0.59) with statistically significant improvements in the tear film break-up time (2.00 versus 1.16, *P* = 0.015) and lissamine green staining (−1.42 versus −0.91, *P* = 0.046). The overall results showed that Cationorm was as safe as, but more effective than, Refresh Tears in patient with mild to moderate DED symptoms.

In a subsequent 3-month, controlled, randomized, single-masked study conducted in Italy, the efficacy of Cationorm was evaluated in adults with moderate dry eye [64]. Seventy-one patients were randomized to treatment with Cationorm, Optive (Allergan), or Emustil (SIFI) 4 times daily, and efficacy assessments were conducted at 1 and 3 months. At 1 month patients treated with Optive and Cationorm experienced a statistically significant improvement from baseline in their dry eye symptoms which was also evident for each of the 3 treatment groups at 3 months. At 3 months, improvements from baseline in the tear break-up time and fluorescein staining were statistically significant for Cationorm and Optive but not for Emustil, and while both Cationorm and Optive significantly reduced tear film osmolarity, only Cationorm showed a statistically significant change compared to Emustil. In this study Cationorm was clearly more effective than Emustil in patients with moderate DED and although not statistically better, the overall improvement in DED symptoms and signs were greater in patients treated with Cationorm than Optive.

The results of the preclinical studies (corneal healing in alkali burn and de-epithelization rabbit models) and clinical trials evaluating Cationorm in patient with DED support its safety and efficacy for the treatment of dry eye symptoms and showed the benefit of the Novasorb cationic emulsion on the ocular surface independent of an active ingredient. However, as we will see, the inherent efficacy of the preservative-free cationic emulsion on improving symptoms of ocular surface disease presented an unanticipated challenge when used as a vehicle in the evaluation of the efficacy of the preservative-free cationic emulsion loaded with CsA in patients with DED.

### 5.2. Clinical Evaluation of Cyclokat

In the DEWS definition of DED it is stated that DED is accompanied by an increased osmolarity of the tear film and inflammation of the ocular surface. As such DED can be considered a chronic, bilateral inflammatory condition for which appropriate treatment, particularly for patients unresponsive to symptomatic treatment with artificial tears would include an anti-inflammatory agent. While Restasis, an anionic emulsion of 0.05% CsA, is available for the treatment of DED in the US, despite the widespread use of hospital compounded CsA and even corticosteroids in the EU there has been no approved pharmaceutical drug indicated for patients with DED. Based on the preclinical data showing the potential advantages of a cationic emulsion over anionic emulsions and unmet medical need for an approved topical CsA formulation in the EU, Novagali undertook the development of Cyclokat for the treatment of dry eye disease.

The initial clinical trial of Cyclokat was a phase II, 3-month, randomized, double-masked, placebo-controlled, dose-ranging study enrolling 53 Gougerot-Sjögren patients with moderate to severe DED. The primary objective of the study was to assess ocular tolerance and systemic safety of the cationic emulsion containing CsA at concentrations of 0.025%, 0.05%, and 0.1% compared to the cationic emulsion vehicle containing no active ingredient. An exploratory evaluation of efficacy was a secondary objective. At baseline, 62% of the enrolled patients had a Schirmer test score of ≤1 mm at 5 minutes and 49% had a corneal fluorescein staining score of ≥3. Over the 3-month treatment period there were no safety concerns and no evidence of systemic absorption of CsA following topical administration of either Cyclokat dose. Patients treated with the 0.1% Cyclokat formulation showed greatest improvements in corneal and conjunctival staining at 3 months and a dose response effect was observed for the reduction of conjunctival HLA-DR staining (a biomarker for ocular surface inflammation) at month 3 compared to baseline (vehicle: −10%; 0.025% CsA: −8%; 0.05% CsA −23%, and 0.01% CsA: −50%).

A second phase II, 3-month, double-masked placebo controlled study comparing Cyclokat 0.05% and 0.1% versus its cationic emulsion vehicle was conducted in 132 patients with mild to moderate DED utilizing the controlled adverse environment chamber. In this study the efficacy and safety of Cyclokat was assessed by the evaluation of coprimary efficacy endpoints (corneal fluorescein staining as the sign and ocular discomfort as the symptom) at month 3 after and during exposure to controlled adverse environment chamber, respectively. Although superiority was not achieved for the coprimary endpoints, there was an overall favorable safety profile and efficacy was demonstrated for the improvement of several secondary endpoints addressing DED signs and symptoms with the results favoring the use of the 0.1% dose for subsequent clinical development.

The Siccanove study was a 6-month phase III, multicenter, randomized, controlled, double-masked trial of Cyclokat 0.1% administered once daily versus its emulsion vehicle in 492 patients with moderate to severe DED. The primary study objective was to demonstrate superiority of Cyclokat on both a DED sign (mean changes in CFS using the modified Oxford scale) and DED symptoms (mean change in global score of ocular discomfort using a VAS). Following a washout period during which only artificial tears were allowed, patients were randomized at baseline to treatment with either Cyclokat (*n* = 242) or its cationic emulsion vehicle (*n* = 250) and evaluated at study visits at months 1, 3, and 6. As early as month 1 (*P* = 0.002), patients treated with Cyclokat showed a statistically significant improvement in the mean change in CFS grade compared to the cationic emulsion vehicle from baseline which continued to improve from month 3 (*P* = 0.030) to month 6, the DED sign coprimary efficacy endpoint. The statistically significant improvements in CFS over 6 months (*P* = 0.009) were complemented by a statistically significant improvement in lissamine green staining (*P* = 0.048) and a reduction in HLA-DR expression (*P* = 0.022) [65]. Additional, post hoc analysis of the Siccanove study data showed that the benefit of treatment with Cyclokat was greatest in patients with the most severe keratitis (as defined by CFS) at baseline (delta in the mean change in CFS from baseline in CFS grade 2–4 = 0.22, *P* = 0.009; 3-4 = 0.32, *P* = 0.005; grade 4 = 0.77, *P* = 0.001) [66]. Although there was a clinically relevant improvement in DED symptoms from baseline the Cyclokat and cationic emulsion vehicle treatment arms, no statistically significant differences were observed at month 6 for the mean change in the global score of ocular discomfort, the DED symptom coprimary efficacy endpoint. However, there was a statistically significant improvement in symptoms for patients achieving a ≥25% improvement in the VAS score (50.21% versus 41.94%, *P* = 0.048). The difficulty in demonstrating the benefit of Cyclokat over its cationic emulsion vehicle was in part attributed to the efficacy of the vehicle itself in improving the symptoms of DED as demonstrated in clinical trials for Cationorm. Additionally, the symptoms coprimary endpoint result can be related to poor correlation between dry eye disease signs and symptoms. At baseline in the Siccanove study, while the mean VAS scores increased with the severity of the CFS, the correlation between the VAS score, as an expression of DED symptoms, and the CFS grade, as an expression of a DED sign, at baseline was low (Spearman's correlation coefficient = 0.23) due to the wide variability in the severity of patient reported symptoms. Similarly at month 6 the statistical correlation between mean change in CFS grade and VAS score was low (Spearman's correlation coefficient = 0.094) with only approximately 68% of patients showing concordance in the direction of change in CFS grade and DED symptoms [65]. Although a poor concordance between dry eye disease signs and symptoms has been recognized in the literature, improvement in both signs and symptoms is an expected outcome in randomized clinical trials investigating new DED treatments. Hence several drugs having shown promise for improving DED have failed due to the inability to demonstrate a statistically significant improvement in signs and symptoms of dry eye disease using coprimary efficacy endpoints.

Fortunately, sign and symptom composite responder endpoints, used in registration trial supporting the approval of new treatments for other chronic inflammatory diseases, provide an alternate method to satisfy the requirement of regulatory authorities. The methodological approach of composite responder analysis avoids issues related to high variability when following mean change of signs and symptoms as discontinuous variables. By focusing only on within-patient's improvements, the composite responder approach could resolve the concern related to the poor correlation between signs and symptoms in evaluating the efficacy of new treatment for DED. As such a pivotal phase III trial, the Sansika study, utilizing a composite responder analysis at month 6, has been initiated to evaluate the efficacy of Cyclokat in patients with severe dry eye disease.

## 6. Conclusion

**Figure 6:**
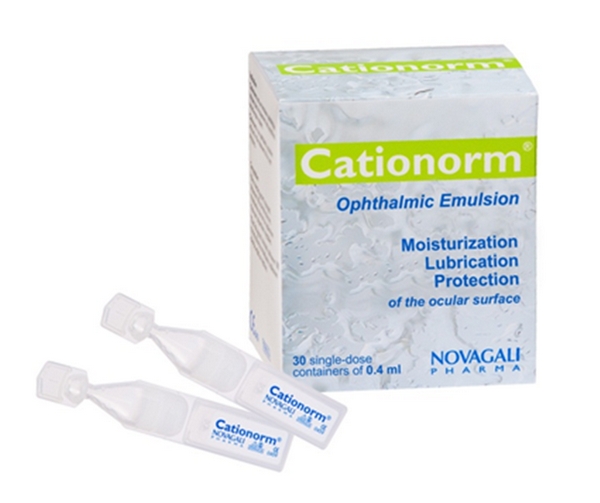
Cationorm is the first product marketed based on the cationic emulsion technology.

Novasorb technology is a typical example of a breakthrough formulation technology primarily developed by an academic team and successfully translated to the patient. Eight years were necessary for the first product to reach the market. With three products in the late stages of clinical development and one product on the market, Novasorb has now proven the concept that cationic nanoemulsions can effectively treat ophthalmic diseases with no toxicity (tested successfully in over 1,000 patients) and several other advantages (Table 10). Cationorm (Figure 6) was launched on the French market April 2008 and at the time this article is written more than 550,000 units of treatment were sold in about 10 countries without any pharmacovigilance concerns. Cyclokat, Vekacia, and Catioprost could reach the market within a few years following the successful completion of pivotal registration studies. The reasons for the success of the Novasorb technology are multiple. Since the beginning of the formulation work, the company prioritized the search for only compoundial and ophthalmology accepted excipients, a manufacturing process which is scalable, and finally the animal models and experimental protocols were designed to carefully screen and select the formulation with the highest probability of demonstrate clinical safety and efficacy.

The Novasorb success story also proves that authorities, particularly European authorities, are relatively open to new delivery approaches and new technologies as long as efficacy and safety can be conclusively demonstrated according to well-constructed protocols and studies. Novagali Pharma is now pursuing the next generation of cationic nanoemulsions, which will have enhanced pharmacokinetics properties and new original drug products to expand the reach of ophthalmic indications. Some other improvements such as development of new cationic agents will provide continued support for this promising and effective means of delivering active molecules.

## Figures and Tables

**Table 1:** The main challenges in ocular drug delivery and key considerations.

*Challenges*	

*Absorption*: only 3 to 4% ocular bioavailability after topical administration with traditional eye drops	
*Poorly-soluble drugs*: conventional aqueous eye drops not suitable for lipophilic drugs (40–60% of new chemical entities)	
*Patient compliance*: multiple instillations are often needed with eye drops to reach therapeutic levels	
*High tolerability/comfort requirements* limit the formulation options	
*Excipient choice*: few excipients listed in ophthalmology (oils, surfactants, polymers…)	
Posterior segment drug delivery: no topical system for the posterior segment; invasive treatments are used due to lack of alternatives	

*Considerations*	

*Anatomy & physiology of the eye*: mucus layer, eyelids, metabolism, blink wash-out…	
*Tear composition*: lipid outer layer, stability of the tear film, enzymes…	
*Disease state*: impact of keratitis or inflammation on absorption and clearance…	
*Ocular comfort*: tolerability of the formulation, pH, osmolality, viscosity, drop size…	
*Patient expectations*: type of packaging and squeeze ability impacting compliance…	
*Drug loading*: impact on absorption, efficacy, dosing regimen, compliance…	

**Table 2:** Chemical structures of common molecules used as cationic agent in drug delivery.

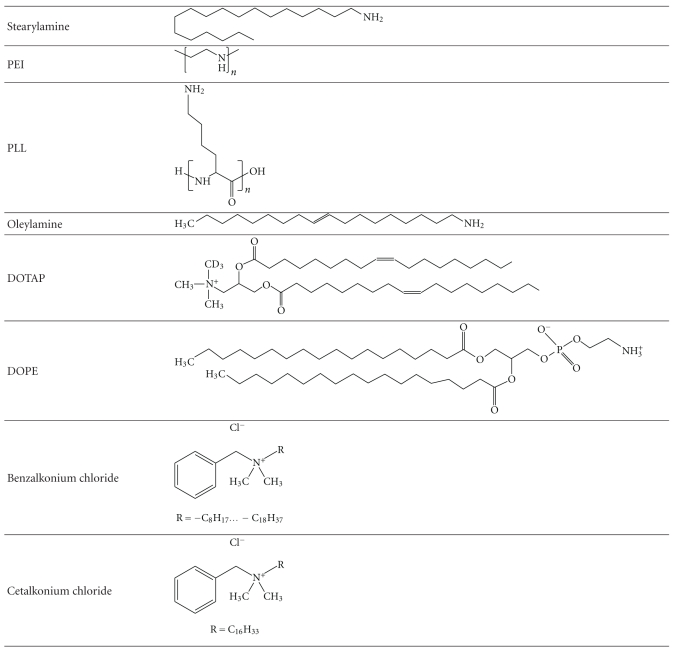

**Table 3:** Excipients which can be used in an ophthalmic emulsion.

Function	Excipients
Osmotic agents	Mannitol, glycerol, sorbitol, propylene glycol, dextrose

Oils	Medium chain triglycerides, mineral oil, vegetal oil such a castor oil

Cationic agents	Benzalkonium chloride, cetylpyridinium chloride, cetrimide, benzethonium chloride

Surfactants	Polysorbates, cremophors, poloxamers, tyloxapol, vitamin E-TPGS

Buffers, salts, and anions	To be avoided if possible

Water	Water for injections

Others	Viscosifying agents: preferably neutral Preservatives: preferably nonionic and hydrophilic

**Table 4:** Final specifications of the cationic nanoemulsions.

Specifications	Values
Aspect	Milky white to translucid
pH	5.5–7
Osmolality	180 to 300 mOsm/kg
Zeta potential	+20 to +40 mV
Mean oil droplet size	150 to 300 nm
Sterility	Sterile
Viscosity	1.1 m²/s
Surface tension	Similar to tears: 41 mN/m

**Table 5:** Composition of a typical vehicle from Novasorb technology.

	Excipients	Function	Concentration % w/w
Oily phase	Medium chain triglyceride	Internal phase	1 to 2
	Cetalkonium chloride	Cationic agent	0.005
	Tylopaxol	Surfactant	0.2

Aqueous phase	Poloxamer 188	Surfactant	0.01
	Glycerol	Osmotic agent	1.5 to 2.5
	NaOH	pH adjuster	Ad pH 6-7
	Water for injections	External phase	Ad 100

**Table 6:** Main product based on Novasorb technology marketed or to be marketed.

Product	Active ingredient	Indication	Status
Cationorm	Medical device	Dry eye	Marketed
Cyclokat	0.1% cyclosporine A	Severe dry eye	Phase III
Vekacia	0.1% cyclosporine A	Vernal keratoconjunctivitis	Phase III
Catioprost	0.005% latanoprost	Glaucoma associated with ocular surface disease	Phase II

**Table 7:** Listing of safety screening and regulatory toxicity studies performed in order to test Novasorb technology in humans.

Nonclinical studies type	Safety studies for Novasorb alone and loaded Novasorb
Safety screening	(i) Draize test
	(ii) Demonstration in a repeated acute rabbit toxicity model that BAK and CKC containing emulsion are well tolerated
	(iii) Ocular safety evaluation of newly developed *in vitro* corneal wound healing model and in an acute *in vivo* rabbit model
	(iv) *In vivo* toxicity evaluation of latanoprost cationic emulsion in the rabbit

Regulatory toxicity studies	(i)* In vitro* evaluation of the cytotoxic potential by indirect contact
	(ii) Delayed-type hypersensitivity evaluation in the Guinea pig
	(iii) Ocular irritation test in the rabbit (short term: 72 h) following a single application
	(iv) Determination of the physical compatibility of Novasorb with contact lenses
	(v) 28-day ocular tolerance in the rabbit
	(vi) Evaluation of the potential to induce delayed contact hypersensitivity (local lymph node assay)
	(vii) Evaluation of the corneal sensitivity following repeated applications in albino rabbits
	(viii) Phototoxicity and photoallergic potential evaluation following topical applications in the Guinea pig
	(ix) 6-month ocular toxicity in the dog and rabbit

**Table 8:** Listing of proof-of-concept and regulatory pharmacokinetics studies performed in order to test Novasorb technology in humans.

Nonclinical studies type	Studies for Novasorb alone and Novasorb loaded
Proof-of-concept	(i) *Ex vivo* measurement of contact angle and surface tension of cationic emulsions on rabbit eyes
	(ii) Evaluation and comparison of the wound healing potential of the cationic emulsion versus artificial tears in a rabbit model of corneal abrasion
	(iii) Evaluation of the efficacy of a 0.1% cyclosporine A cationic emulsion in the management of keratoconjunctivitis sicca in the dog
	(iv) Evaluation of the efficacy of a cationic emulsion of 0.005% latanoprost at reducing elevated intraocular pressure in glaucomatous monkeys
	(v) *In vitro* and *in vivo* evaluation of a preservative-free cationic emulsion of latanoprost in corneal wound healing models

Regulatory pharmacokinetics studies	(i) Single and multiple doses pharmacokinetic
	(ii) Systemic exposure determination and toxicokinetics following repeated instillations of BAK and CKC-containing cyclosporine A cationic emulsion

**Table 9:** Clinical trials performed with Novasorb.

Year	Phase type	Product	Objectives	Indication	No. of patients
2003	Phase I	Vehicle no.1	Tolerance and safety	None	16
2004	Phase II		Tolerance and safety, Exploratory efficacy	Dry eye	50
2005	Phase II	Cationorm (Vehicle no.2)	Efficacy, tolerance, and safety	Dry eye	79
2010	Phase II		Efficacy, tolerance, and safety	Dry eye	71
2005	Phase IIa	Cyclokat	Tolerance and safety Exploratory efficacy	Dry eye disease	48
2008	Phase IIb		Exploratory efficacy, tolerance, and safety	Dry eye disease	132
2009	Phase III “Siccanove”		Efficacy, tolerance, and safety	Dry eye disease	496
2011	Phase III “Sansika”		Efficacy, tolerance, and safety	Dry eye disease	252
2006	Phase IIb/III	Vekacia	Efficacy, tolerance, and safety	Active VKC	118
2009	Phase IIb		Efficacy, tolerance, and safety	Nonactive VKC	34
2011	Phase II	Catioprost	Exploratory efficacy, open-label study	Glaucoma	NA
2011	Phase IIb		Exploratory efficacy, tolerance, and safety	Glaucoma	100

VKC: Vernal keratoconjunctivitis.

**Table 10:** Key drivers of cationic emulsion technology Novasorb.

(i) Solubilization of large doses of lipophilic drugs and/or large molecules	
(ii) Better penetration through membranes resulting in enhanced bioavailability	
(iii) Potential for drug controlled release	
(iv) Stable and can be sterilized	
(v) Addition of effective novel routes of administration to existing marketed drugs	
(vi) Expanding markets and indications	
(vii) Extending product life cycles	
(viii) Generating new opportunities	
(ix) Inexpensive to manufacture	
